# The Space Between: Navigating Dementia Care as a Trans/Nonbinary Elder in a Polarized World

**DOI:** 10.1002/alz70858_096706

**Published:** 2025-12-24

**Authors:** Dáithí Clayton

**Affiliations:** ^1^ DEEP Network, Aalter, Aalter, Belgium

## Abstract

At 70+ years old, I live with early‐onset dementia—a journey marked by both profound loss and a fierce determination to preserve my identity. As a trans/nonbinary person, my diagnosis carries a unique weight: the relentless search for safe, affirming medical and dementia care in systems that are increasingly shaped by anti‐gender ideologies and heteronormativity. This is not merely a personal fight for dignity, but a broader call to action at a time when extreme right‐wing political currents are taking hold across the USA, Canada, Europe, and beyond, erasing the hard‐won rights and visibility of trans and nonbinary people.

In this presentation, I will share my lived experience navigating care settings where my gender identity is often misunderstood, ignored, or erased. I will explore the challenges of finding inclusive palliative and dementia care—places where I am seen, respected, and where my queerness is celebrated to the very end of my life. I will speak to the fear that lingers for so many of us: the risk of being forced back into closets we fought to escape, surrounded by caregivers who may view our identities as an inconvenience or as something to correct. I will also highlight the urgent need for intersectional, LGBTQI+‐affirming dementia care models that center the voices of trans/nonbinary elders, particularly as our communities face increasing hostility.

This work is not theoretical; it is survival. As more of us age into care systems ill‐prepared to meet our needs, the stakes could not be higher. Through this deeply personal narrative, Daithi aims to offer hope, provoke reflection, and inspire action. Dementia care must be a safe place for all—inclusive, affirming, and free from the corrosive influence of anti‐gender ideologies. My journey is a testament to resilience and a plea to care professionals, researchers, and policymakers: do not erase us. Instead, work alongside us to create spaces where all identities can thrive, even as memory fades.

Key themes: LGBTQI+ inclusion in dementia care, trans/nonbinary lived experience, intersectionality, palliative care, and combating anti‐gender ideologies

